# Decoupling Sugar Availability and Metabolic Efficiency Reveals Bottlenecks in Ethanol Production from Lignocellulosic Hydrothermal Hydrolysates

**DOI:** 10.1021/acsomega.6c07430

**Published:** 2026-10-06

**Authors:** Francisco D. Vezaro, Alex Schulz, Barbara V. da Rosa, Chrysttian Matheus Schneider Garcia, Raquel C. Kuhn, José A. C. Teixeira, Ederson R. Abaide, Flávio D. Mayer

**Affiliations:** † Laboratory of Biomass and Biofuels, 28118 Federal University of Santa Maria, Roraima Avenue, 97105-900 Santa Maria, Brazil; ‡ Department of Chemical Engineering, Federal University of Santa Maria, Roraima Avenue, 97105-900 Santa Maria, Brazil; § Centre of Biological Engineering, 450509 University of Minho, Campus de Gualtar, 4710-057 Braga, Portugal

## Abstract

Lignocellulosic hydrolysates derived from hydrothermal processing often result in low ethanol yields, which are commonly attributed to inhibitory compounds and suboptimal sugar composition. However, the relative contribution of sugar availability and microbial metabolic efficiency to these limitations remains poorly understood. This study systematically evaluated the ethanol production by *Scheffersomyces stipitis* using hydrolysates obtained from steam explosion (SE), subcritical water hydrolysis (SWH), enzymatic hydrolysis (EH), and their sequential combinations, under identical fermentation conditions. To decouple process limitations, ethanol yields were analyzed based on both total sugar available and sugar effectively consumed. Results showed that hydrothermal hydrolysates (SE and SWH) led to low ethanol yields (0.08–0.14 g/g), primarily due to limited sugar uptake associated with the presence of inhibitory compounds and low sugar concentrations. In contrast, enzymatic hydrolysates, particularly after SE pretreatment (SE + EH), achieved yields up to 0.50 g/g, approaching the theoretical maximum. Notably, when yields were calculated based on sugar consumed, significantly higher efficiencies were observed across all conditions, revealing that the intrinsic metabolic capacity of *S. stipitis* remained largely preserved even in inhibitory media. These findings demonstrate that reduced ethanol production in lignocellulosic hydrolysates is predominantly governed by limitations in sugar assimilation rather than metabolic conversion. This study provides a mechanistic framework for distinguishing between substrate accessibility and metabolic efficiency, offering new insights for the optimization of second-generation ethanol processes.

## Graphical abstract

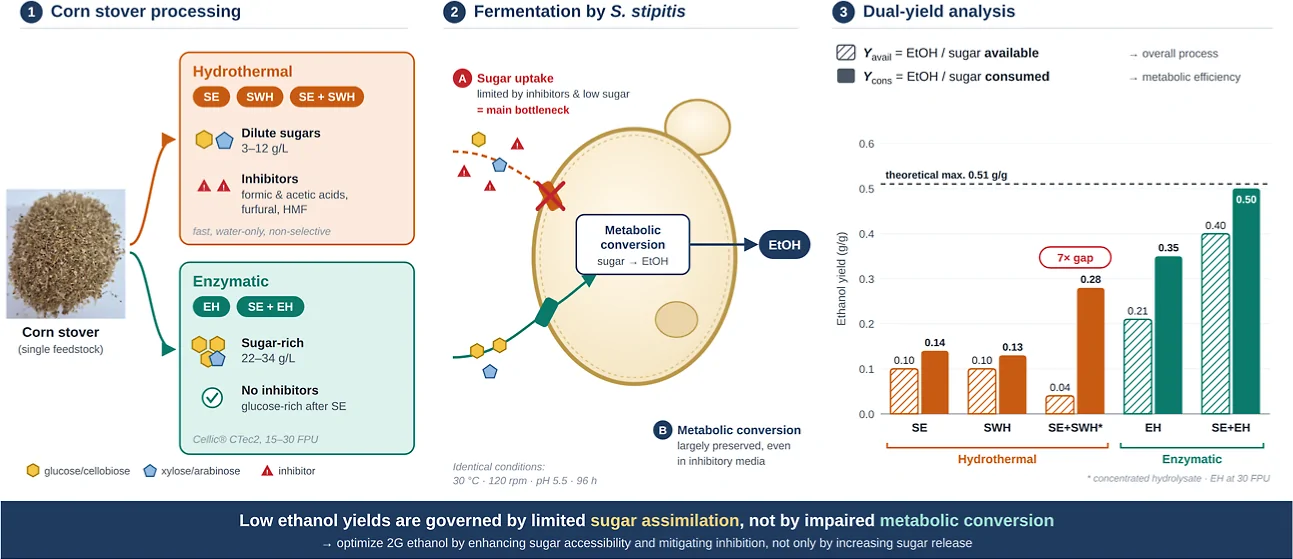

## 1 Introduction

The high availability of lignocellulosic residues makes their utilization for the production of value-added products of significant socioeconomic interest.^1^ Because of their complex composition, these materials represent an abundant source of fermentable sugars derived mainly from cellulose and hemicellulose. Among the possible valorization routes, fermentation of these sugars to produce ethanol remains one of the most established and promising pathways.^2^

Corn stover, including stalks, leaves, husks, and cobs remaining after grain harvest, represents one of the most abundant lignocellulosic agricultural residues worldwide. Global corn stover production is estimated to be approximately 1.28 billion tonnes annually. In Brazil, the production of approximately 140 million tonnes of corn grain corresponds to an estimated 140 million tonnes of corn residues per year, assuming a stover-to-grain ratio of 1:1 on a dry matter basis.^3^ However, processing corn stover for conversion into fermentable sugars and subsequently into ethanol requires biomass pretreatment to further optimize these still low yields.^4^ In recent years, steam explosion (SE) and subcritical water hydrolysis (SWH) have received increasing attention as promising pretreatment strategies for lignocellulosic biomass due to their reduced chemical requirements and potential integration into biorefinery schemes.^5^ Numerous studies have focused on optimizing the operating conditions to maximize sugar release or minimize inhibitor formation. However, these processes are often evaluated independently, using different biomasses, microbial strains, and fermentation conditions, which limits direct comparison of their impact on downstream fermentation performance.^6^

SWH, in particular, has gained increasing attention as a rapid (few minutes) and environmentally benign processing method for lignocellulosic materials, capable of hydrolyzing both cellulose and hemicellulose.^7^ Nevertheless, due to its non-selective nature, SWH can promote parallel degradation reactions, leading to the formation of inhibitory compounds. As a result, hydrolysates obtained via SWH remain comparatively underexplored with respect to their applicability in ethanol fermentation, whereas sugars derived from enzymatic hydrolysis (EH) have been more extensively investigated.^8^

Despite the growing body of literature on individual pretreatment and hydrolysis strategies, comparative studies assessing the fermentation performance of hydrolysates obtained from different processing routes under identical biological conditions remain scarce. In particular, few studies systematically evaluate SE, SWH, and EH, either alone or in combination, using the same lignocellulosic feedstock and the same fermenting microorganism.^9,10^ Such integrated comparisons are essential to identifying the key bottlenecks associated with sugar availability, inhibitor formation, and microbial assimilation in realistic second-generation ethanol processes.

In this context, *Scheffersomyces stipitis* represents a suitable model microorganism for evaluating lignocellulosic hydrolysates, as it is one of the few naturally occurring yeasts capable of fermenting both hexose and pentose sugars to ethanol without genetic modification.^11,12^ This yeast exhibits high ethanol yields from pentoses such as xylose and arabinose under oxygen-limited conditions while producing low levels of byproducts, including xylitol and acetic acid.^13^ Moreover, *S. stipitis* shows moderate tolerance to common lignocellulosic inhibitors, such as acetic acid, furfural, and hydroxymethylfurfural (HMF), which are typically present in hydrolysates generated by hydrothermal and subcritical water pretreatments.^14^ Rather than focusing exclusively on maximizing ethanol titers, the use of *S. stipitis* enables the assessment of how different hydrolysis routes influence sugar uptake, metabolic efficiency, and fermentation limitations under inhibitory conditions.

Therefore, this study aims to systematically evaluate ethanol production from hydrolysates obtained via SE, SWH, EH, and their sequential combinations using a single lignocellulosic feedstock and a consistent fermentation system based on *S. stipitis*. Beyond a conventional comparison of processing routes, this work introduces a dual-yield analytical approach in which ethanol yields are interpreted based on both total sugar availability and the fraction of sugars effectively consumed. This framework enables the decoupling of limitations associated with substrate accessibility from those related to the intrinsic metabolic efficiency. Therefore, the present study seeks to provide mechanistic insights into the primary bottlenecks governing ethanol production from lignocellulosic hydrolysates, contributing to a more rational design and optimization of second-generation biorefinery systems.

## 2 Materials and Methods

### 2.1 Raw Material Preparation and Characterization

Corn stover was obtained from experimental crops at the Universidade Federal de Santa Maria, Brazil after grain harvesting. The entire corn stover was subjected to drying and grinding, followed by sieving to obtain a particle size between 16 and 32 mesh. Afterward, it was stored at −5 °C until being analyzed for cellulose, hemicellulose, and lignin composition according to the methodology proposed by the National Renewable Energy Laboratory.^15^ Subsequently, the biomass underwent SE, SWH, and EH assays.

The yeast *S. stipitis* ATCC 58376 was purchased from Fundação André Tosello, Campinas, Brazil, and *Saccharomyces cerevisiae* was obtained in lyophilized form from LNF Latino Americana, Bento Gonçalves, Brazil. Both were stored at 4 °C until further use.

### 2.2 Fermentation Procedures

#### 2.2.1 Inoculum Preparation

The inoculum preparation and fermentations were performed in duplicate in Erlenmeyer flasks using the yeast *S. stipitis*. The procedures for cultivation and proliferation were performed based on previous studies, with some adaptations.^16,17^ The materials and solutions used for cultivation and fermentation were sterilized in an autoclave at 121 °C for 20 min. Subsequently, all materials were handled in a laminar flow chamber sterilized by exposure to 70% (v/v) ethanol and UV light.

The cultivation of *S. stipitis* was performed on Petri dishes using a substrate containing 40 g/L glucose, 10 g/L peptone, and 15 g/L agar, incubated at 28 °C for 7 days. To evaluate cell growth in the inoculum, assays were conducted in duplicate using media with either 20 g/L glucose or 20 g/L xylose, supplemented with 10 g/L peptone and 10 g/L yeast extract. For this purpose, 250 mL of the substrate was placed in Erlenmeyer flasks, and a portion of the culture obtained from the Petri dishes was added. This mixture was incubated in a shaker (Shaker supplier: Tecnal, Model: TE-420) at 30 °C and 120 rpm for 48 h. Samples of 15 mL were collected at 0, 6, 12, 18, 24, 30, and 48 h. The samples were transferred to tubes and centrifuged at 4000 rpm for 10 min (Centrifuge supplier: Eppendorf, Model: 5804 R), the supernatant was removed, and the cell pellets were dried in an oven at 60 °C for subsequent weighing. The cell quantity in the inoculum was then calculated using eq 1:

1 $$C_{CE}=\frac{m_{tube\;after}-m_{tube\;before}}{V_{ino}}$$

where *C*_CE_ is the cell concentration (g/L), *m*_tubeafter_ is the mass of the tube after drying with the cells, *m*_tubebefore_ is the mass of the empty tube, and *V*_ino_ is the volume of the centrifuged inoculum (0.015 L). Based on this, the inoculum containing 20 g/L glucose, 10 g/L peptone, and 10 g/L yeast extract was selected.

#### 2.2.2 Fermentation of the Synthetic Substrate

After cultivating *S. stipitis* in the inoculum for 24 h, 30 mL of the inoculum was transferred to tubes and centrifuged at 4000 rpm for 10 min (centrifuge supplier: Eppendorf, Model: 5804 R), and the supernatant was removed. The cells present in the 30 mL inoculum were then used for each of the fermentation assays. The assays reported in Table 1 were thus replicated. The sugar concentrations presented in Table 1 were selected to provide representative levels of fermentable sugars that may be present in lignocellulosic hydrolysates and to enable the evaluation of the metabolic behavior of the yeast strains under controlled conditions.^18^ The inclusion of C5 sugars in the *S. cerevisiae* assay was intentional, as the same mixed-sugar environment was used to directly compare the substrate utilization and metabolic responses of both yeasts rather than to assume inefficient pentose metabolism by *S. cerevisiae*.

**Table 1:** Fermentation Assays in the Synthetic Substrate

assay	glucose (g/L)	xylose (g/L)	cellobiose (g/L)	arabinose (g/L)	yeast
A1	23.4 ± 1.6	0.0 ± 0.0	0.0 ± 0.0	0.0 ± 0.0	*Scheffersomyces stipitis*
A2	0.0 ± 0.0	19.7 ± 1.0	0.0 ± 0.0	0.0 ± 0.0	*Scheffersomyces stipitis*
A3	12.3 ± 0.3	7.9 ± 0.1	0.0 ± 0.0	0.0 ± 0.0	*Scheffersomyces stipitis*
A4	9.0 ± 0.1	11.6 ± 0.0	0.0 ± 0.0	0.0 ± 0.0	*Scheffersomyces stipitis*
A5	5.4 ± 0.1	14.6 ± 0.4	0.0 ± 0.0	0.0 ± 0.0	*Scheffersomyces stipitis*
A6	10.9 ± 0.0	0.0 ± 0.0	11.8 ± 0.1	0.0 ± 0.0	*Scheffersomyces stipitis*
A7	10.5 ± 0.1	0.0 ± 0.0	0.0 ± 0.0	10.0 ± 0.1	*Scheffersomyces stipitis*
A8	20.1 ± 0.1	19.0 ± 0.1	12.5 ± 0.1	11.5 ± 0.8	*Scheffersomyces stipitis*
A9	20.7 ± 0.0	20.4 ± 0.0	11.9 ± 0.1	10.9 ± 0.6	Saccharomyces cerevisiae

For each assay, 100 mL of the fermentation substrate (Table 1) and 1.3 g/L of cells were used and incubated in a shaker (shaker supplier: Tecnal, Model: TE-420) at 120 rpm and 30 °C. Samples were collected at 0, 6, 12, 24, 36, 48, 72, and 96 h for subsequent analysis by high-performance liquid chromatography (HPLC). Fermentations with *S. cerevisiae* followed the same protocol as those with *S. stipitis*, with the exception of the inoculation procedure. In this case, 1.3 g/L of commercially obtained lyophilized *S. cerevisiae* was directly added to the substrate, without any prior cultivation step.

#### 2.2.3 Fermentation of Hydrolysates Obtained by Hydrothermal Processing of Corn Stover

The SE assays were conducted using 11.25 g of corn stover at 200 °C with a residence time of 10 min (based on the conventional severity factor log *R*_0_ = 3.94).^19^ The semi-continuous SWH assays were performed at temperatures of 230 and 260 °C, with water-to-biomass ratios of 20 and 40, for 30 min. Additionally, combinations involving SE followed by sequential SWH (SE-CS hydrolysis) were performed, integrating SE–SWH processing. These hydrolysates were obtained as described in a previous study^7^ and stored at −5 °C for later use. Additionally, the concentrated substrates shown in Table 2 underwent an evaporation step conducted at 105 °C, reducing the total volume by 50–70%.

**Table 2:** Conditions for Obtaining Substrates through Hydrothermal Processing

	temperature (°C)	water-to-biomass ratio
SE	200	9
CS hydrolysis	230	40
SE-CS hydrolysis	230	40
SE^a^	200	9
CS hydrolysis^a^	260 and 230	20 and 40
SE-CS hydrolysis^a^	260 and 230	20 and 40

a SE (steam explosion), CS hydrolysis (untreated corn stover hydrolysis), and SE-CS hydrolysis (hydrolysis of corn stover treated by steam explosion). The samples were concentrated by evaporation in an oven at 105 °C.

Fermentation of the hydrolysates obtained through hydrothermal processing was carried out similarly to that described in Section 2.2.2. However, the pH of hydrolysates was adjusted from approximately 3.5 to 5.5 using ACS grade calcium carbonate in order to neutralize the excess organic acids present in the substrate, produced during the hydrothermal processing of corn stover.

#### 2.2.4 Fermentations of Hydrolysates Obtained by EH of Corn Stover

The EH of corn stover was performed based on previous studies, with some adaptations.^20^ For this purpose, a commercial cellulolytic enzyme cocktail (Cellic CTec2) was used, presenting an experimentally determined activity of 60 FPU/mL, as measured by the standard filter paper assay according to National Renewable Energy Laboratory recommendations.^21^ EH assays were conducted using 5 g of untreated corn stover or pretreated corn stover by SE at 200 °C for 10 min. The experiments were performed with a solvent-to-biomass ratio of 20, using sodium citrate buffer (0.05 M, pH 4.8) and the Cellic CTec2 enzymatic cocktail at enzyme loadings of 15 and 30 FPU (equivalent to 3 FPU/g and 6 FPU/g of biomass, respectively). Each flask was incubated in an orbital shaker (shaker supplier: Tecnal, model: TE-420) at 250 rpm and 50 °C for 48 h.

Subsequently, the fermentation assays were performed similarly to those described in Section 2.2.3, with pH adjusted to 5.5 to standardize conditions in relation to the hydrothermal processing fermentation assays. It is important to note that the corn stover remained in the Erlenmeyer flasks throughout the fermentation process, eliminating the need for a solid–liquid separation step.

### 2.3 Characterization of Hydrolysates and Fermentations

The concentrations of sugars, ethanol, organic acids, and inhibitors were determined using HPLC, employing a Shimadzu Proeminence UFLCXR system (Model LC-2050C 3D), following a previously established methodology. The concentrations of cellobiose, glucose, xylose, arabinose, acetic acid, formic acid, and ethanol were quantified using a Hi-Plex H chromatographic column (300 mm × 7.7 mm, Agilent, Santa Clara, California, USA) coupled with a refractive index detector (RID-20A). To determine the concentrations of furfural and 5-hydroxymethylfurfural (HMF), a Shim-Pak ODS 115 C18 column (Shimadzu, Kyoto, Kansai, Japan) and a photodiode array detector (PDA 20-A, Shimadzu, Japan) were used.

The ethanol production rate (g/L h) was calculated by eq 2, while the yield ethanol/sugar (g/g) was determined by eq 3:

2 $$R_{et}=\frac{C_{et}}{t_{n}-t_{n-1}}$$

3 $$Y_{g/g}=\frac{C_{et}}{C_{g}}$$

where *R*_et_ is the ethanol production rate over the fermentation time (g/L.h), *C*_et_ is the concentration of ethanol at the time of collection (g/L), *t*_*n*–1_ is the time elapsed from the collection of the previous fermentation to the time of the current collection (h), e *t*_*n*_ is the time of the current collection (h), *Y*_g/g_ is the yield ethanol/sugar (g/g), and *C*_g_ is the sum of glucose, xylose, arabinose, cellobiose concentration of the hydrolysate from SE, SWH, SE + SWH, EH, and SE + EH.

The ethanol yield compared to the maximum theoretical yield (theoretical yield (%)) was calculated by the eq 4:

4 $$T_{y}=\frac{Y_{g/g}\cdot 100}{0.51}$$

where *T*_y_ is the theoretical yield (%) and 0.51 is the maximum theoretical ethanol yield per mass of sugar.^29^

### 2.4 Statistical Analysis

The Tukey test was performed using statistical software (StatSoft Inc., Palo Alto, CA, USA) to assess significant differences at a 95% confidence level (*p* ≤ 0.05) in the concentrations of fermentable sugars, organic acids, inhibitors, and ethanol following the hydrolysis and fermentation assays.

## 3 Results and Discussion

In addition to fermentation assays conducted in a synthetic medium to assess the performance of *S. stipitis* under approximately ideal conditions, other substrates obtained through different processing routes of corn stover were also evaluated. The lignocellulosic hydrolysates analyzed in this study, derived from SE, EH, SWH, and the combinations of SE + EH and SE + SWH, differ substantially in their chemical compositions and inhibitory profiles, which directly influence the fermentation behavior of *S. stipitis*.

SE is an established pretreatment that promotes extensive hemicellulose solubilization and partial lignin disruption; however, the elevated temperatures and acid conditions typically lead to the formation of degradation products, such as acetic acid, furfural, and 5-hydroxymethylfurfural (HMF). Furthermore, SE can promote the formation of pseudolignin, particularly under severe pretreatment conditions, through the condensation and redeposition of lignin-derived and carbohydrate degradation products on the biomass surface.^7^ This pseudolignin-rich layer can increase the non-productive adsorption of cellulases and partially block their access to cellulose, thereby reducing EH efficiency and sugar release. Consequently, additional delignification, such as alkaline treatment, may be required to improve enzyme accessibility and increase fermentable sugar yields.^18^ Since the amount of sugars available for fermentation directly affects ethanol production, these structural limitations can ultimately influence the overall fermentation performance. However, the scope of the work focused on evaluating the effects of hydrothermal and enzymatic processes on the hydrolysates and fermentation, without the use of chemicals.

SWH is an approach that uses only water under high temperatures (230 and 260 °C), resulting in the non-selective hydrolysis, leading to the release of hexoses, pentoses, organic acids, and degradation compounds derived from the breakdown of cellulose and hemicellulose. EH can result in a substrate rich in hexoses and pentoses, depending on the enzymatic cocktail employed. In this study, since cellulolytic and hemicellulolytic enzymes were used, the process tended to yield exclusively sugars derived from cellulose and hemicellulose, without the formation of organic acids or inhibitory compounds.

SE tends to remove a significant portion of the hemicellulose from the biomass, consequently increasing the relative amount of cellulose. In this context, it is expected that after SE pretreatment, the hydrolysates subsequently obtained by SWH would present a composition mainly derived from cellulose, including glucose, cellobiose, formic acid, and HMF, with lower proportions of compounds originating from hemicellulose. The EH step, when applied after pretreatment by SE, mainly converts the remaining cellulose into fermentable glucose with minimal inhibitor generation, producing a cleaner hydrolysate that favors microbial growth and ethanol production. Therefore, comparing the fermentation performance across these hydrolysates provides valuable insights into how *S. stipitis* responds to different substrate chemistries, sugar compositions, and inhibitor levels, highlighting its adaptability and limitations under real biorefinery conditions.

### 3.1 Inoculum Preparation

*S. stipitis* is capable of assimilating both glucose and xylose, as reported in the literature.^22^ In this context, the yeast’s cultivation for inoculum preparation may vary depending on the sugar used. The behavior of *S. stipitis* in terms of sugar consumption, ethanol production, and cell growth is shown in Figure 1, with glucose as the carbon source in panel (A) and xylose in panel (B). Glucose consumption was completed within 18 h of fermentation. In contrast, xylose was not fully consumed, decreasing from 20.8 g/L to 1.6 g/L after 30 h and to 1.4 g/L after 48 h of fermentation, confirming the yeast’s ability to assimilate xylose but to a lesser extent compared to glucose.

**Figure 1:**
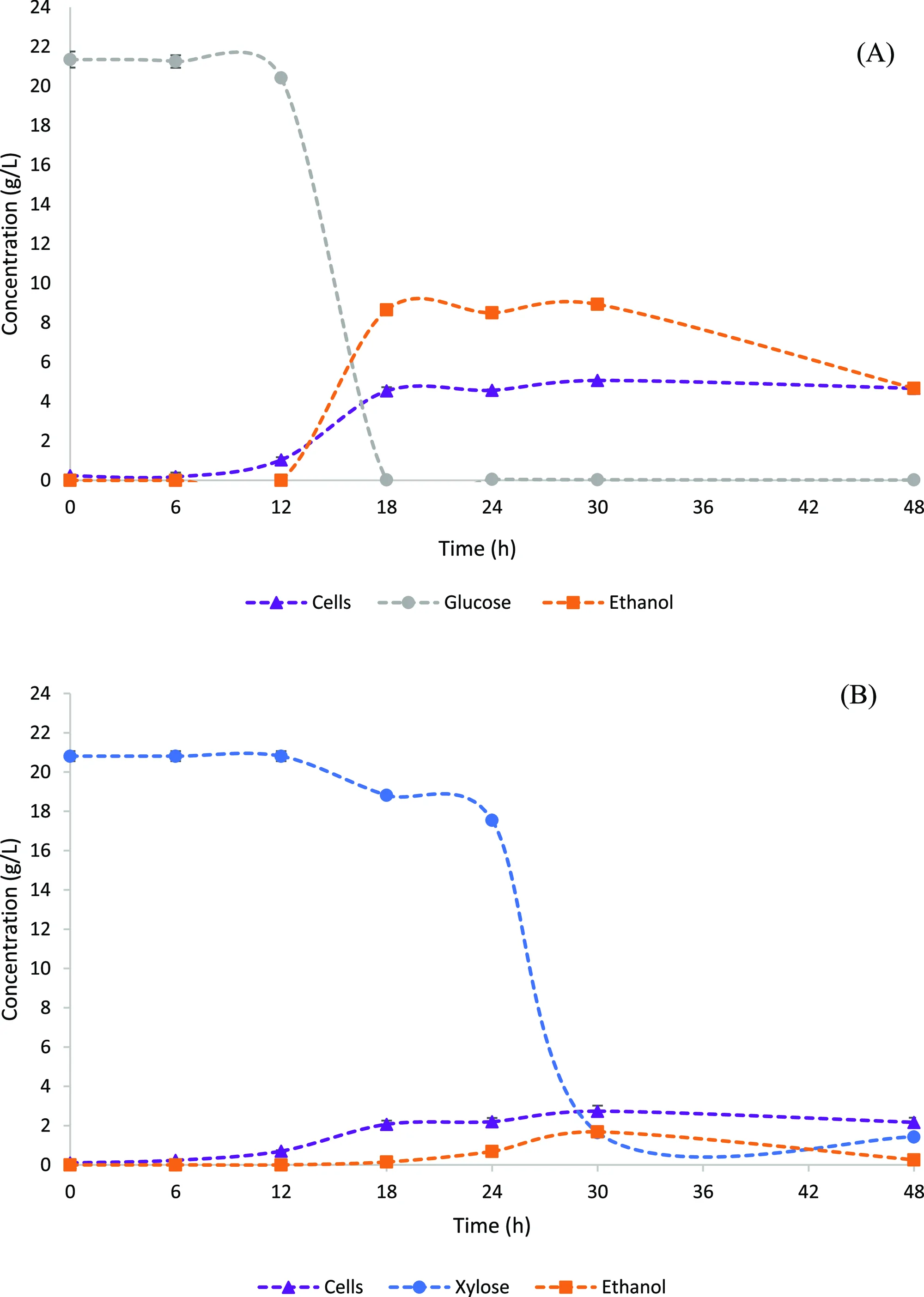
Cell growth profile of *Scheffersomyces stipitis* during the consumption of glucose (A) and xylose (B).

Ethanol production in both assays showed a considerable difference; 9.0 g/L ethanol was obtained in the presence of glucose, whereas only approximately 1.7 g/L was produced using xylose. However, ethanol production during the inoculum preparation is not the main objective. Notably, *S. stipitis* exhibited superior cell growth when glucose was used as the carbon source. Cell concentrations of 4.5, 4.6, and 5.0 g/L were achieved after 18, 24, and 30 h of fermentation, respectively, with glucose in the substrate. In contrast, when xylose was used, the cell concentrations were 2.0, 2.2, and 2.7 g/L at the same period. On the basis of these results, glucose was selected as the sugar for inoculum preparation, with a fermentation time of 24 h to avoid maintaining cells for an extended period without nutrient access.

The longer time required for *S. stipitis* to consume xylose may be related to the greater complexity of xylose metabolism compared to glucose. Xylose undergoes reduction to xylitol by the enzyme xylose reductase and subsequent oxidation of xylitol to xylulose by xylitol dehydrogenase, which is then phosphorylated to xylulose-5-phosphate. In contrast, glucose is directly phosphorylated to glucose-6-phosphate.^23^ Another factor that may influence cell growth and sugar consumption is the yeast’s capacity to transport xylose across the plasma membrane into the cell, with glucose transport occurring more readily.^24^ Thus, due to the higher cell proliferation observed, glucose was selected as the sugar for inoculum preparation for subsequent fermentations.

### 3.2 Fermentation of the Synthetic Substrate

Lignocellulosic biomass can provide different types of sugars derived from cellulose and hemicellulose, mainly glucose and xylose. In this context, experiments using different combinations of glucose, xylose, arabinose, cellobiose, and *S. stipitis* were conducted, as shown in Table 3. Furthermore, the ethanol yield was evaluated using two complementary approaches: based on the total sugar available in the hydrolysate and based on the fraction of sugars effectively consumed by the yeast. The yield based on available sugar reflects the overall performance of the process, considering both sugar availability and subsequent conversion to ethanol, whereas the yield based on consumed sugar specifically reflects the efficiency of ethanol formation from the carbon sources assimilated by yeast. This distinction is particularly relevant for complex lignocellulosic hydrolysates, in which incomplete sugar assimilation may occur due to inhibitors, mixed sugar compositions, or preferential substrate utilization.^25^ Therefore, the combined analysis of both metrics allows limitations associated with substrate availability and uptake to be distinguished from those associated with the fermentative conversion of assimilated sugars.

**Table 3:** Fermentation Performance of *Scheffersomyces stipitis* on Synthetic Sugar-Rich Media Designed to Mimic the Composition of Hydrolysates Derived from Different Lignocellulosic Biomasses

assay	sugars	glucose (g/L)	secondary sugar (g/L)	ethanol yield (g/L)	yield ethanol/sugar available (g/g)	yield ethanol/sugar consumed (g/g)^a^	theoretical yield based on sugar available (%)	theoretical yield based on sugar consumed (%)^a^
A1	glucose	23.4 ± 1.6	0.0 ± 0.0	8.9 ± 0.7^a^	0.38 ± 0.00^a^	0.38 ± 0.00^a^	74.5 ± 0.6^a^	75.04 ± 0.82^a^
A2	xylose	0.0 ± 0.0	19.7 ± 1.0	4.8 ± 0.0^c^	0.24 ± 0.00^d^	0.25 ± 0.00^b,c^	47.6 ± 0.2^d^	48.94 ± 0.03^b,c^
A3	glucose/xylose	12.3 ± 0.3	7.9 ± 0.1	7.3 ± 0.7^a,b^	0.36 ± 0.03^a,b^	0.39 ± 0.00^a^	71.0 ± 6.1^a,b^	76.41 ± 0.68^a^
A4	glucose/xylose	9.0 ± 0.1	11.6 ± 0.0	6.3 ± 0.1^b,c^	0.31 ± 0.01^b,c^	0.31 ± 0.01^a,b^	63.1 ± 1.0^b,c^	61.73 ± 1.06^a,b^
A5	glucose/xylose	5.4 ± 0.1	14.6 ± 0.4	5.7 ± 0.1^c^	0.28 ± 0.00^c,d^	0.30 ± 0.00^b^	55.9 ± 0.4^c,d^	58.38 ± 0.58^b^
A6	glucose/cellobiose	10.9 ± 0.0	11.8 ± 0.1	2.4 ± 0.4^d^	0.10 ± 0.02^e^	0.18 ± 0.04^c,d^	20.6 ± 3.5^e^	35.93 ± 7.14^c,d^
A7	glucose/arabinose	10.5 ± 0.1	10.0 ± 0.1	1.8 ± 0.0^d^	0.09 ± 0.00^e^	0.17 ± 0.00^d^	17.5 ± 0.1^e^	32.71 ± 0.98^d^

a Different letters in the same column indicate that the means differ significantly by the Tukey test (*p* ≤ 0.05). Ethanol yield based on sugar consumed until the ethanol production peaks.

Examining Table 3, using glucose as the sole carbon source, it was possible to convert 23.4 ± 1.6 g/L glucose into 8.9 ± 0.7 g/L ethanol, resulting in a yield of 0.38 ± 0.00 g/g sugar available. In contrast, when 19.7 ± 1.0 g/L xylose was used, only 4.8 ± 0.0 g/L ethanol was produced, with a yield of 0.24 ± 0.00 g/g sugar available. These results indicate that although the yeast is capable of metabolizing xylose, glucose is more efficiently converted into ethanol.

When evaluating fermentations using different proportions of glucose and xylose, assay A3 showed an ethanol production of 7.3 ± 0.7 g/L, with a yield of 0.36 g/g sugar available. In assays A1 and A2, the ethanol/sugar available yields were 0.38 g/g and 0.24 g/g, respectively. Considering that A3 contained 12.3 g/L glucose and 7.9 g/L xylose, if the sugars were converted with the same efficiencies observed in A1 and A2, the expected ethanol production would be approximately 6.6 g/L (4.7 g/L from glucose and 1.9 g/L from xylose). This value corresponds to the lower limit of ethanol yield observed in A3, considering the standard deviation. Nonetheless, this suggests that the combination of sugars may enhance the overall conversion of carbohydrates into ethanol, as evidenced by the increased average yield of 7.3 ± 0.7 g/L. A similar trend was observed in assays A4 and A5, although to a lesser extent, indicating that a higher glucose concentration may improve xylose conversion into ethanol.

Biomass can also generate other sugars that may be utilized during fermentation, such as cellobiose, due to incomplete cellulose hydrolysis, and arabinose, which is found in hemicellulose. When fermentations were performed using cellobiose and arabinose in combination with glucose (assays A6 and A7), ethanol yields were lower than those expected from glucose alone, as observed in assay A1, resulting in a theoretical yield of 20.6 ± 3.5% for A6 and 17.5 ± 0.1% for A7. In assays A6 and A7, the glucose concentrations were 10.9 and 10.5 g/L, respectively, which could theoretically result in approximately 4 g/L ethanol based solely on the 0.38 g/g sugar available yield observed in A1. This suggests that under the tested fermentation conditions, the yeast was not able to convert arabinose and cellobiose into ethanol. Additionally, these sugars may require energy derived from glucose for their assimilation, redirecting cellular metabolism toward alternative pathways that do not result in ethanol production.^26^

The highest yield compared to the theoretical maximum was observed when using glucose as the only carbon source (74.5 ± 0.6%). An increase in the xylose proportion in the fermentation assays led to a progressive decrease in theoretical yield, which reached 71.0 ± 6.1% (A3), 63.1 ± 1.0% (A4), 55.9 ± 0.4% (A5), and 47.6 ± 0.2% (A2). A potential explanation for the reduced ethanol yield is the diversion of xylose metabolism toward xylitol production by the yeast.^27^ In a study using 20 g/L glucose fermented by *S. stipitis*, an ethanol concentration of approximately 7.5 g/L was reported, corresponding to a yield of 0.37 g/g sugar available and 73.5% of the theoretical maximum.^16^ Under controlled aeration conditions, ethanol yields ranging from 0.34 g/g sugar available to 0.41 g/g sugar available were reported using 20 to 25 g/L xylose, with fermentation times between 100 and 180 h,^28^ which may explain the higher conversion efficiency from xylose. In another study, a yield of 0.30 g/g sugar available was reported with 84% sugar consumption.^29^

In Table 3, the ethanol yield calculated based on sugar consumed provides a more accurate assessment of the fermentative performance of *S. stipitis*, as it excludes the fraction of sugars that remained unassimilated during fermentation. While ethanol yields based on total sugar available ranged from 0.09 to 0.38 g/g, the yields based on sugar consumed were consistently higher, particularly in assays containing mixed sugars. For example, assays A3 and A4 (glucose/xylose mixtures) reached ethanol yields of up to 0.39 g/g based on sugar consumed, corresponding to theoretical yields above 75%*. This indicates that once sugars were effectively taken up by the yeast, their conversion to ethanol occurred with high metabolic efficiency. In contrast, substrates containing cellobiose or arabinose (A6 and A7) showed low yields based on the sugar available. However, the yield based on sugar consumed nearly doubled, highlighting that the main limitation in these assays was sugar uptake rather than fermentative metabolism. These results reinforce the importance of distinguishing between sugar availability and sugar utilization when evaluating yeast performance, especially on complex or nonpreferred substrates.

Figure 2 shows the ethanol yield profile as a function of fermentation time for assays A1 to A7, as presented in Table 3. When using glucose as the sole carbon source, the maximum ethanol yield was reached within 12 h of fermentation, with complete sugar consumption in the same period. In contrast, when using only xylose, this pentose was consumed more slowly, reaching a residual concentration of 0.6 g/L after 72 h, with an ethanol yield of 4.8 g/L.

**Figure 2:**
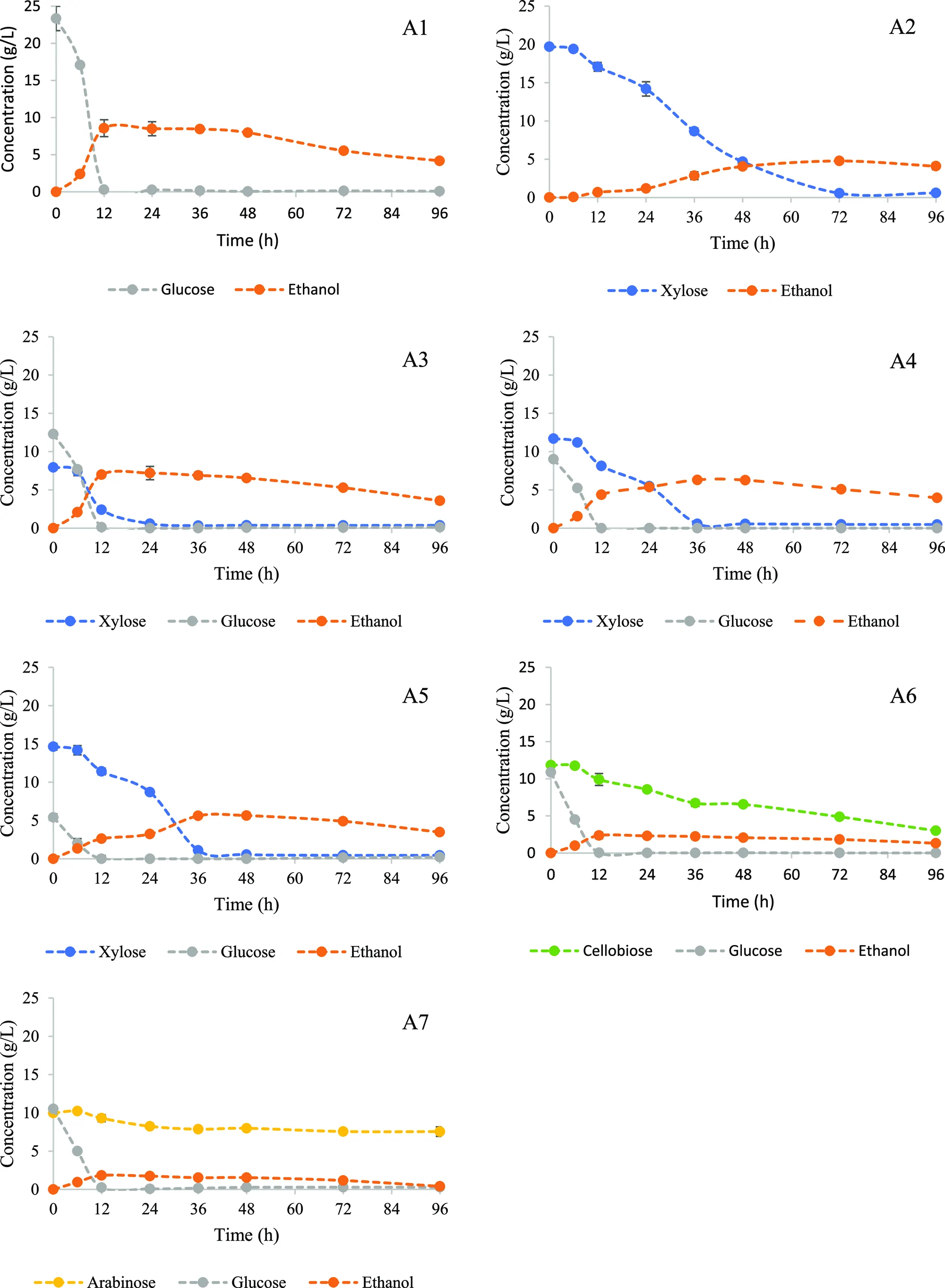
Fermentation profile of *Scheffersomyces stipitis* in different synthetic substrates. (A1) 23.4 ± 1.6 g/L glucose; (A2) 19.7 ± 1.0 g/L xylose; (A3) 12.3 ± 0.3 g/L glucose and 7.9 ± 0.1 g/L xylose; (A4) 9.0 ± 0.1 g/L glucose and 11.6 ± 0.0 g/L xylose; (A5) 5.4 ± 0.1 g/L glucose and 14.6 ± 0.4 g/L xylose; (A6) 10.9 ± 0.0 g/L glucose and 11.8 ± 0.1 g/L cellobiose; and (A7) 10.5 ± 0.1 g/L glucose and 10.0 ± 0.1 g/L arabinose.

When mixing different proportions of glucose and xylose, the time to achieve the maximum concentration of ethanol is higher with the higher concentration of xylose and the lower concentration of glucose in the substrate. Specifically, assays A3, A4, and A5 showed peak ethanol production at 24 h (7.2 g/L), 36 h (6.3 g/L), and 48 h (5.7 g/L), respectively. In all three assays, glucose was completely consumed within 12 h. In A3, xylose was almost fully consumed after 24 h (0.6 g/L remaining), in A4, it was gradually consumed up to 36 h (0.6 g/L), and in A5, it was reduced to 0.5 g/L only after 36 h, matching the time at which the maximum ethanol yield was reached in each case. Several factors may explain why xylose is consumed more slowly and yields less ethanol than does glucose. Xylose requires additional metabolic steps for assimilation, such as its conversion to xylitol and then to xylulose, and often results in byproducts like xylitol and acetate. Moreover, the transport of xylose across the plasma membrane may be less efficient than that of glucose.^24^

In assay A6, which used glucose and cellobiose, it was observed that the yeast could gradually assimilate the disaccharide. Some studies have shown that fungi of the genus *Pichia* are capable of producing cellulolytic and hemicellulolytic enzymes after 1 to 3 days of fermentation,^27^ which may indicate a significant advantage for this type of yeast in lignocellulosic fermentations, especially considering the potential presence of oligosaccharides resulting from incomplete hydrolysis of the lignocellulosic matrix. In this context, it is possible that *S. stipitis* was able to hydrolyze cellobiose into glucose and subsequently assimilate the monosaccharide.

The ethanol production in A6 was lower compared to other assays using glucose and xylose, possibly due to the yeast channeling cellobiose into alternative metabolic pathways, converting it to glucose via intracellular β-glucosidases and directing nutrients toward respiration and cell maintenance.^30^ Another study testing *S. stipitis* with cellobiose in fermentation reported no ethanol production from the disaccharide, although resveratrol was obtained as a product.^31^ In another case, using cellobiose as the carbon source, an ethanol/sugar yield of only 0.06–0.09 g/g was observed.^32^ Additionally, other studies have reported the yeast’s ability to produce xylanase,^33^ xylitol,^27^ and succinic acid^34^ under different fermentation conditions.

Assay A7, which used glucose and arabinose, showed the lowest ethanol yield among all of the seven assays. Additionally, the yeast consumed only 26.5% of the available arabinose, indicating a low affinity for the metabolism of this monosaccharide. The ethanol production peak occurred at 12 h of fermentation because of glucose consumption. Similar to what was observed in assay A6 with cellobiose, arabinose may have negatively influenced ethanol production from glucose. Similar results have been reported in fermentations involving xylose and arabinose mixtures,^35^ as well as with glucose, xylose, and arabinose, where *S. stipitis* failed to consume arabinose, while xylose and glucose were fully assimilated.^36^

The fermentation assays showed a trend of decreasing ethanol concentration in the absence of nutrients or in the presence of sugars that were less preferred by the yeast. Ethanol consumption by *S. stipitis* itself has already been reported in the literature under aerobic conditions, especially when carbon sources are absent or limited.^17^ In addition, due to the extended fermentation time, partial ethanol volatilization is also possible.^37^

#### 3.2.1 Comparison between *S. stipitis* and *S. cerevisiae* in the Fermentation of Different Fermentable Sugars

*S. stipitis* is of particular interest for the fermentation of hydrolysates derived from lignocellulosic biomass because of its ability to assimilate various sugars beyond glucose. In this context, to evaluate the advantages and challenges of using *S. stipitis* (assay A8) in comparison to the traditional *S. cerevisiae* (assay A9), which is widely used for industrial ethanol production from glucose, fermentations were conducted with both yeasts using glucose, xylose, cellobiose, and arabinose, as shown in Table 4.

**Table 4:** Evaluation of Sugar Consumption and Ethanol Production by *Scheffersomyces stipitis* and *Saccharomyces cerevisiae* Synthetic Sugar-Rich Substrates Representative of Those Obtained from Different Lignocellulosic Biomasses

assay	yeast	cellobiose (g/L)	glucose (g/L)	xylose (g/L)	arabinose (g/L)	total sugars (g/L)
A8	*Scheffersomyces stipitis*	12.5 ± 0.1	20.1 ± 0.1	19.0 ± 0.1	11.5 ± 0.8	63.1 ± 1.0
A9	Saccharomyces cerevisiae	11.9 ± 0.1	20.7 ± 0.0	20.4 ± 0.0	10.9 ± 0.6	63.9 ± 0.6
	yeast	ethanol yield (g/L)	yield ethanol/sugar available (g/g)	yield ethanol/sugar consumed (g/g)^a^	theoretical yield based on sugar available (%)	theoretical yield based on sugar consumed (%)^a^
A8	*Scheffersomyces stipitis*	18.1 ± 0.4	0.29 ± 0.01	0.36 ± 0.03	56.39 ± 2.08	71.46 ± 6.35
A9	Saccharomyces cerevisiae	9.3 ± 0.0	0.15 ± 0.00	0.39 ± 0.00	28.66 ± 0.23	77.21 ± 0.14

a Ethanol yield based on sugar consumed until the ethanol production peaks.

Based on assays A1 and A2 from Table 3, the conversion of glucose and xylose into ethanol by *S. stipitis* was 0.38 g/g and 0.24 g/g, respectively. Accordingly, in the fermentation presented in Table 4, with higher concentrations of glucose and xylose, it would be possible to obtain 12.2 g/L ethanol from these sugars. Alternatively, considering the conversions observed in A3 and A4 (glucose and xylose mixtures), with yields of 0.36 g/g and 0.31 g/g, the expected ethanol concentrations would be 14.1 g/L and 12.1 g/L, respectively. In this context, the observed production of 18.1 g/L ethanol using all four sugars suggests that, depending on the reference yields (from A1–A2 or A3–A4), between 4.0 and 6.0 g/L ethanol may have originated from the metabolism of cellobiose or arabinose. On the other hand, considering the theoretical maximum stoichiometric yield of 0.51 g/g from glucose and xylose alone, it would be possible to achieve up to 19.9 g/L ethanol.

*S. cerevisiae*, incapable of metabolizing sugars other than glucose into ethanol, produced 9.3 g/L ethanol from a glucose concentration of 20.7 g/L. Thus, considering glucose alone, it exhibited an ethanol/sugar yield of 0.45 g/g, demonstrating a superior capacity to convert glucose into ethanol compared with *S. stipitis*, which showed a yield of 0.38 g/g (Table 3). However, considering the presence of other sugars derived from biomass, *S. stipitis* produced 94.6% more ethanol than *S. cerevisiae* because of its ability to metabolize different sugars into ethanol. Nevertheless, it is noteworthy that after 12 h of fermentation, *S. cerevisiae* produced 9.3 g/L ethanol with a sugar consumption of 23.7 g/L, corresponding to a yield of 0.39 g/g. *S. stipitis* produced up to 18.1 g/L ethanol after 60 h of fermentation with a sugar consumption of 50.1 g/L, resulting in a yield of 0.36 g/g.

Considering the total sugars, approximately 63 g/L sugars (glucose, xylose, arabinose, and cellobiose) were utilized. Accordingly, the ethanol/sugar yields were 0.29 g/g and 0.15 g/g, corresponding to theoretical yields of 59.39% and 28.66% for *S. stipitis* and *S. cerevisiae*, respectively. *S. stipitis* is known for its ability to metabolize glucose and xylose into ethanol. However, in these experiments, approximately 10 g/L cellobiose and arabinose were also present. These sugars showed lower conversion efficiency into ethanol, which explains the lower yields compared with the theoretical maximum. If one considers that the ethanol produced by *S. stipitis* originated exclusively from glucose and xylose, then an ethanol/sugar yield of 0.46 g/g and a theoretical yield of 90.95% would have been achieved. However, these values exceed those reported in Table 3 for fermentations using only glucose and xylose, suggesting that part of the ethanol may have been produced from the metabolism of cellobiose and/or arabinose.

The comparison presented in Table 4 clearly demonstrates the relevance of ethanol yield based on sugar consumed for interpreting yeast performance in sugar-rich substrates. Although *S. cerevisiae* (A9) exhibited a lower ethanol yield when calculated on the basis of total sugar available (0.15 g/g), its yield based on sugar consumed reached 0.39 g/g*, corresponding to more than 77%* of the theoretical maximum. This reflects the high fermentative efficiency of *S. cerevisiae* toward the sugars it is able to metabolize, while a substantial fraction of sugars (pentoses) remained unconsumed. Conversely, *S. stipitis* (A8) showed a higher ethanol yield based on sugar available due to its broader substrate range but a lower yield based on sugar consumed compared to *S. cerevisiae*. These results emphasize that yield calculations based solely on sugar availability may underestimate the intrinsic metabolic efficiency of yeasts and overestimate the performance of pentose-fermenting strains when sugar uptake is incomplete.

Figure 3 shows the profiles of sugar consumption and ethanol production and ethanol production rates by *S. stipitis* and by *S. cerevisiae* over 96 h of fermentation. Throughout this period, *S. stipitis* assimilated 92.7% of the sugars, whereas *S. cerevisiae* assimilated 46.8%. Glucose was completely consumed by both yeasts within the first 12 h, during which *S. stipitis* produced 8.3 g/L ethanol and *S. cerevisiae* produced 9.3 g/L, corresponding to ethanol production rates of 0.82 g/L·h and 0.99 g/L·h, respectively. Additionally, a slight decrease in xylose concentration was observed after 6 h of fermentation. After glucose depletion, ethanol production by *S. cerevisiae* ceased. Nevertheless, minor reductions in xylose and arabinose concentrations were observed up to 96 h, suggesting that *S. cerevisiae* may have attempted to metabolize these sugars, although inefficiently.

**Figure 3:**
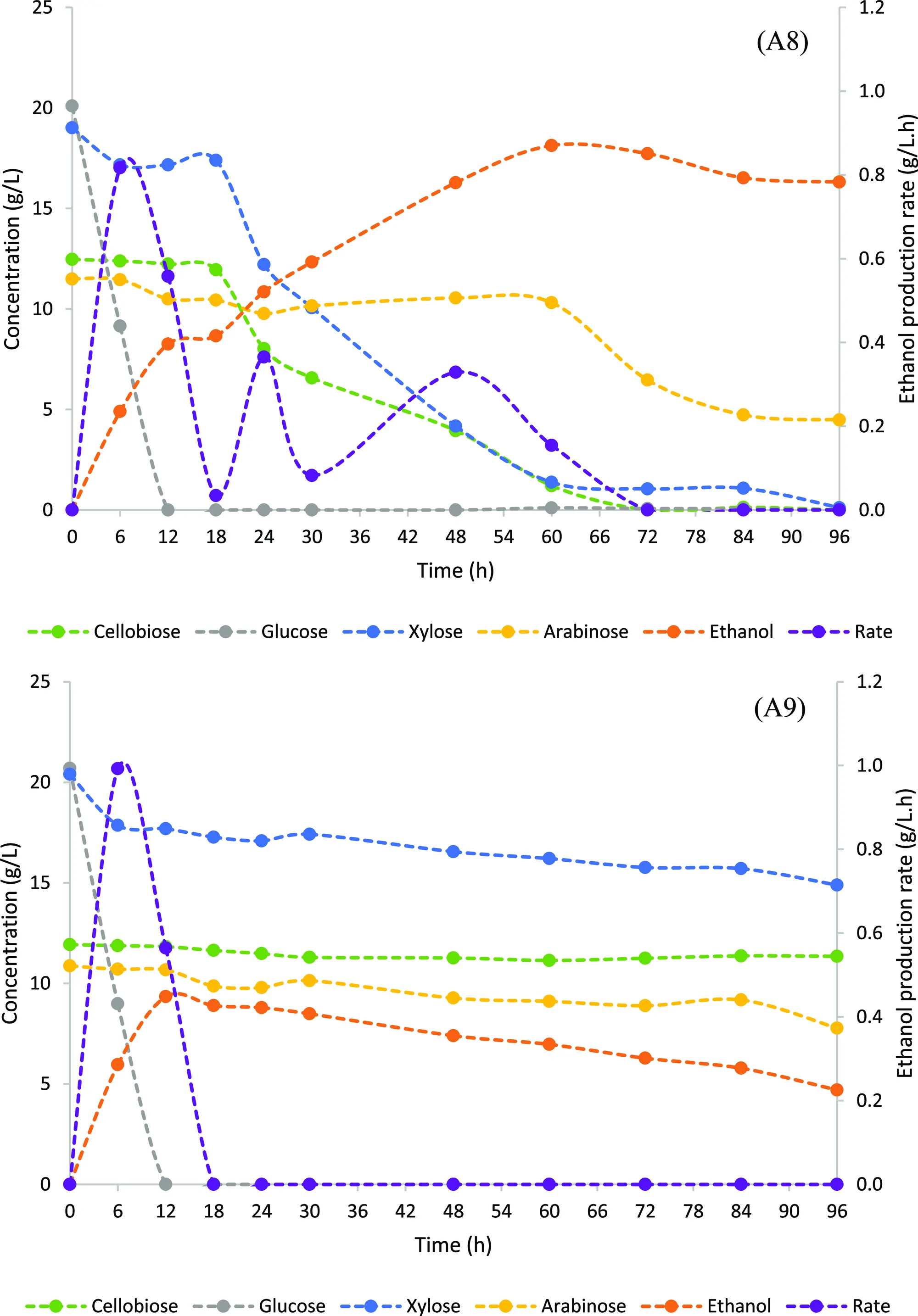
Fermentations using *Scheffersomyces stipitis* (A8) and *Saccharomyces cerevisiae* (A9) in sugar-rich substrates derived from different biomass sources.

The sampling conducted after 18 h of fermentation indicated that neither yeast strain consumed additional sugars between 12 and 18 h, resulting in low ethanol production during this interval. *S. stipitis* exhibited an ethanol production rate of only 0.03 g/L·h during this period. This stagnation likely reflects a metabolic adaptation phase following the depletion of glucose, which had been preferentially assimilated. Between 18 and 60 h of fermentation, xylose and cellobiose were gradually consumed, reaching residual concentrations of 1.4 g/L and 1.2 g/L, respectively, leading to a total ethanol production of 18.1 g/L by 60 h. *S. stipitis* only began to assimilate arabinose to a greater extent after the depletion of the other three sugars, reaching 4.5 g/L by 96 h. However, no additional ethanol production was observed during this period.

Between 12 and 60 h of fermentation with *S. stipitis*, the ethanol concentration increased from 8.3 g/L to 18.1 g/L (an increment of 9.8 g/L). During this interval, glucose was no longer present in the substrate, and the consumption of 17.6 g/L xylose and 11.0 g/L cellobiose by the yeast was observed. Considering the theoretical maximum stoichiometric yield of 0.51 g/g, this amount of xylose could produce up to 9.0 g/L ethanol. Based on the conversion yields from assays A2 (0.24 g/g), A3 (0.36 g/g), and A4 (0.31 g/g) in Table 3, ethanol production from xylose alone would be estimated at 4.2 g/L, 6.3 g/L, and 5.5 g/L, respectively. Thus, between 4.2 g/L and 9.0 g/L ethanol produced from 12 to 60 h may have originated from xylose, indicating that between 0.8 g/L and 5.7 g/L was produced through the metabolism of cellobiose during the same period. These results suggest that under conditions of higher sugar availability, unlike assay 6 from Table 3 (which used 10.9 g/L glucose and 11.8 g/L cellobiose), *S. stipitis* may be capable of efficiently converting cellobiose into ethanol. This enhanced conversion could be associated with the increased energetic availability, potentially leading to a more efficient fermentation of this disaccharide, especially when considering the simultaneous consumption of xylose and cellobiose.

### 3.3 Fermentation of Corn Stover Hydrolysates Obtained by Hydrothermal Processing

SE and semicontinuous SWH are capable of breaking down biomass polysaccharides and releasing sugars,^7^ which can be metabolized by *S. stipitis*. However, these hydrothermal processes still face some challenges, such as the low sugar concentration due to the high water-to-biomass ratio and the degradation of products, including the formation of organic acids and fermentation inhibitors.^38,39^ Despite these disadvantages, they are fast processing methods, capable of fully hydrolyzing biomass within 5 to 30 min, and are considered environmentally friendly.^40^ Therefore, further studies are needed to optimize the use of hydrolysates produced by these technologies.

Previous experiments involving SE, SWH, and SWH of corn stover pretreated by SE were conducted.^7^ The sugars and platform chemicals obtained from these hydrothermal processing methods are presented in Table 5. In experiments A13 to A15, the hydrolysates underwent a concentration step by evaporation to assess the yeast fermentation performance under higher concentrations of sugars, organic acids, and fermentation inhibitors. Experiments A10 to A12 showed sugar concentrations ranging from 3.07 g/L to 4.88 g/L, organic acids from 0.16 g/L to 0.64 g/L, and inhibitors from 0.06 g/L to 1.00 g/L. In contrast, experiments A13 to A15 had total sugar concentrations between 4.88 g/L and 12.11 g/L, organic acids ranging from 1.76 g/L to 4.10 g/L, and inhibitors ranging from 0.63 g/L to 1.55 g/L.

**Table 5:** Fermentation Performance of *Scheffersomyces stipitis* Using Hydrolysates Obtained by SE, SWH of Untreated Corn Stover, and SWH of Steam-Exploded Corn Stover

assay	method of obtaining the substrate	cellobiose (g/L)	glucose (g/L)	xylose (g/L)	arabinose (g/L)	formic acid (g/L)	acetic acid (g/L)	furfural (g/L)	HMF (g/L)	ethanol (g/L)	yield ethanol/sugar available (g/g)	yield ethanol/sugar consumed (g/g)^a^	theoretical yield based on sugar available (%)	theoretical yield based on sugar consumed (%)^a^
A10	SE	1.69 ± 0.32^a^	0.70 ± 0.15^c^	1.00 ± 0.20^c^	1.48 ± 0.8^b^	0.21 ± 0.01^d^	0.42 ± 0.01^d^	0.10 ± 0.01^c^	0.30 ± 0.02^b^	0.49 ± 0.04^a^	0.10 ± 0.02^a^	0.14 ± 0.01^a,b^	20.64 ± 4.48^a^	26.53 ± 1.37^a,b^
A11	CS hydrolysis	1.63 ± 0.01^a^	0.90 ± 0.00^c,d^	0.75 ± 0.00^c^	0.77 ± 0.00^b^	0.19 ± 0.04^d^	0.27 ± 0.01^e^	0.06 ± 0.07^c^	0.00 ± 0.00^c^	0.50 ± 0.04^a^	0.10 ± 0.02^a^	0.13 ± 0.00^b^	20.64 ± 4.48^a^	26.12 ± 0.18^b^
A12	SE-CS hydrolysis	1.72 ± 0.21^a^	0.50 ± 0.01^d^	0.35 ± 0.04^d^	0.48 ± 0.08^b^	0.11 ± 0.01^d^	0.04 ± 0.01^f^	0.06 ± 0.01^c^	0.94 ± 0.12^a^	0.26 ± 0.04^a^	0.08 ± 0.02^a,b^	0.12 ± 0.05^b^	16.87 ± 4.65^a,b^	24.33 ± 8.84^b^
A13^b^	SE^b^	2.08 ± 0.40^a^	2.53 ± 0.13^a^	4.49 ± 0.13^a^	3.02 ± 0.03^a^	2.49 ± 0.06^a^	1.64 ± 0.06^a^	0.65 ± 0.05^b^	0.02 ± 0.01^b^	0.41 ± 0.00^a^	0.03 ± 0.00^b^	0.13 ± 0.00^b^	6.61 ± 0.33^b^	24.53 ± 0.8^b^
A14^b^	CS hydrolysis^b^	1.44 ± 0.01^a^	1.62 ± 0.02^b^	0.59 ± 0.03^c.d^	1.22 ± 0.02^b^	1.16 ± 0.05^c^	0.59 ± 0.01^b^	1.55 ± 0.17^a^	0.00 ± 0.00^b^	0.45 ± 0.15^a^	0.09 ± 0.03^a,b^	0.13 ± 0.02^b^	18.14 ± 5.63^a,b^	24.66 ± 3.97^b^
A15^b^	SE-CS hydrolysis^b^	2.07 ± 0.02^a^	1.72 ± 0.00^b^	1.70 ± 0.02^b^	1.29 ± 0.01^b^	1.32 ± 0.03^b^	0.90 ± 0.01^c^	0.63 ± 0.01^b^	0.02 ± 0.00^b^	0.29 ± 0.03^a^	0.04 ± 0.01^b^	0.28 ± 0.07^a^	8.34 ± 1.03^b^	54.83 ± 14.45^a^

a Ethanol yield based on sugar consumed until the ethanol production peaks.

b The samples were concentrated by evaporation in an oven at 105 °C. Different letters in the same column indicate that the means differ significantly by the Tukey test (*p* ≤ 0.05).

No significant variations in ethanol yield were observed among the six assayed conditions, which may indicate that, even under higher sugar concentrations, the increased presence of organic acids or inhibitors likely impaired the yeast’s ability to produce ethanol. The highest ethanol/sugar yields were found in experiments 1 (SE) and 2 (SWH), both reaching 0.10 g/g, corresponding to 20.64% of the theoretical maximum yield. It is important to note that, in addition to the presence of organic acids and fermentation inhibitors, many of the sugars included in the yield calculation, such as arabinose and, to some extent, cellobiose, were not efficiently converted into ethanol.

For the hydrothermal hydrolysates shown in Table 5, ethanol yields based on sugar consumed were systematically higher than those based on sugar available, confirming that incomplete sugar consumption played a major role in limiting overall ethanol production. In non-concentrated hydrolysates (A10–A12), the yields based on sugar consumed ranged from 0.12 to 0.14 g/g*, corresponding to theoretical efficiencies between 24 and 27%*. Although these values remain low, they indicate that once sugars were assimilated, their conversion to ethanol was moderately efficient despite the presence of inhibitory compounds such as furfural, HMF, and organic acids. In concentrated hydrolysates (A13–A15), the discrepancy between the two yield metrics became more pronounced, particularly in assay A15, where the ethanol yield based on sugar consumed reached 0.28 g/g* (54.8%* of the theoretical yield), while the yield based on sugar available remained below 0.05 g/g. This suggests that fermentation inhibition was primarily associated with reduced sugar uptake rather than impaired ethanol biosynthesis, reinforcing the importance of using sugar-consumed-based yields when evaluating inhibitory hydrolysates.

Figure 4 (A10) corresponds to the fermentation of the liquid fraction obtained from SE of corn stover at 200 °C for a 10 min residence time. It can be observed that the majority of the available sugars are cellobiose, due to incomplete cellulose hydrolysis, and arabinose and xylose, which derive from the less thermoresistant hemicellulose. Xylose and cellobiose were fully consumed after 48 and 72 h, respectively, whereas glucose and arabinose were not entirely consumed. Ethanol production reached 0.3 g/L after 6 h. However, by 48 h, the ethanol concentration dropped to 0 g/L, then rose again, reaching 0.5 g/L after 96 h. In addition to the low sugar concentrations during fermentation, the presence of HMF and furfural may have hindered ethanol production as well as glucose assimilation.^14^

**Figure 4:**
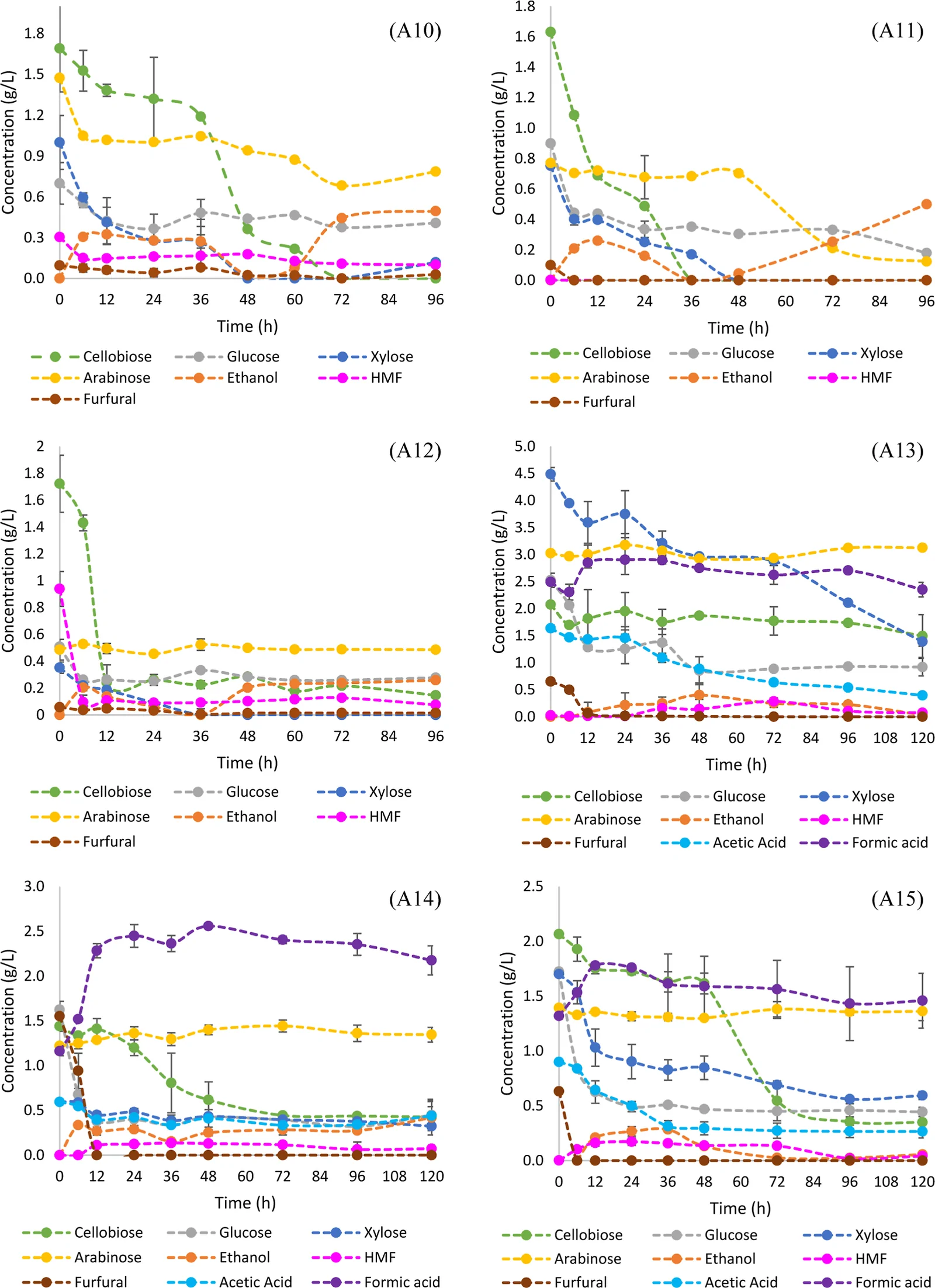
Fermentation profiles of hydrolysates obtained from different hydrothermal treatments of corn stover using *Scheffersomyces stipitis*. (A10) Hydrolysate obtained by SE; (A11) hydrolysate obtained by SWH of untreated corn stover; (A12) hydrolysate obtained by SE + SWH. Panels (A13), (A14), and (A15) correspond to the same processing routes as (A10), (A11), and (A12), respectively, but produced under higher-severity operating conditions and subjected to an additional concentration step by evaporation prior to fermentation, resulting in increased sugar and inhibitor concentrations. Each panel shows the time course of sugar consumption, ethanol production, and the evolution of inhibitory compounds during fermentation.

Sugar consumption and ethanol production during the fermentation of the substrate obtained by SE are shown in Figure 4 (A10). In this substrate, cellobiose and xylose were completely assimilated, whereas only 41.7% of glucose and 46.7% of arabinose were consumed relative to their initial concentrations. Figure 4 (A11) corresponds to the SWH of untreated corn stover, where sugar consumption reached 92.5% of the total. In this assay, both cellobiose and xylose were fully consumed, glucose was reduced by 84.4%, and arabinose by 81.0%, resulting in an ethanol concentration of 0.5 g/L after 96 h of fermentation. In Figure 4 (A12), sugar consumption was 70.6%, with xylose fully consumed, cellobiose and glucose reduced by 91.5 and 45.4%, respectively, while arabinose was not assimilated, achieving an ethanol concentration of approximately 0.3 g/L. It is noteworthy that Figure 4 (A12) exhibited a greater concentration of HMF (0.94 g/L), corresponding to the assay with the lowest sugar consumption, followed by the SE assay in Figure 4 (A10) with 0.30 g/L HMF and 0.10 g/L furfural, and lastly, Figure 4 (A11) with 0.10 g/L furfural.

Figure 4 (A13–A15) corresponds to fermentations of other hydrolysates subjected to a concentration step by evaporation. Since the concentration step significantly increased the levels of formic acid and acetic acid, these compounds were monitored during fermentation. It can be observed that the ethanol yield was lower compared to the nonconcentrated hydrolysates, possibly due to the higher presence of acids as well as furfural, which inhibited sugar metabolism by the yeast. The sugars were not fully assimilated, with consumption rates of 42.8, 47.9, and 60.1% for Figure 4 (A13–A15), respectively. Accordingly, the arabinose concentration remained constant in all three assays, while cellobiose, glucose, and xylose were partially consumed.

Alcoholic fermentation of substrates obtained by SWH has scarcely been addressed in the literature. However, fermentation of SWH hydrolysates from soybean straw and soybean hulls using the yeast *Wickerhamomyces* sp. reported ethanol concentrations of 0.69 g/L and 0.72 g/L, respectively. The soybean straw hydrolysate contained 2.16 g/L glucose, 1.33 g/L xylose, 0.08 g/L arabinose, 4.76 g/L formic acid, 8.22 g/L acetic acid, 0.28 g/L HMF, and 0.48 g/L furfural. For soybean hulls, concentrations of 0.96 g/L glucose, 1.11 g/L xylose, 0.43 g/L arabinose, 0.09 g/L cellobiose, 3.24 g/L formic acid, 3.14 g/L acetic acid, 0.16 g/L HMF, and 0.31 g/L furfural were observed.^9^ Similar results were reported for the fermentation of substrates obtained solely by SWH from sugarcane bagasse after concentration by rotary evaporation. Using a consortium of *S. cerevisiae* and *S. stipitis*, less than 1 g/L ethanol was produced, along with incomplete xylose consumption.^41^

In the assays using concentrated substrates obtained from hydrothermal processes, an increase in formic acid concentration was observed between 6 and 12 h of fermentation. This period coincided with significant sugar consumption by yeast. The most plausible explanation for the production of formic acid is that the fermentation conditions were unfavorable, leading the yeast to activate the alternative metabolic pathways. Similar cases have been reported during fermentation with *S. cerevisiae* when sodium chloride was added to the substrate, where the addition of sodium chloride to the substrate led to an increase in formic acid production from 1.8 g/L to 11.9 g/L.^42^

As shown in Figure 4, most of the assays presented an initial concentration of furfural or HMF, which decreased within the first 6 to 12 h of fermentation. In some cases, after the reduction of furfural, a slight increase in the HMF concentration was observed. The literature reports that certain bacteria and fungi are capable of biotransforming HMF into 5-hydroxymethyl-furan-2-carboxylic acid and 5-formyl-2-furancarboxylic acid and furfural into 2-furoic acid and furfuryl alcohol.^43,44^ Furthermore, it has been reported that *S. stipitis* can assimilate up to 6 g/L acetic acid during fermentation, although this results in reduced sugar consumption and inhibited ethanol production,^45^ which is consistent with the results of the present study.

The effects of some inhibitors in synthetic media, such as HMF, furfural, and acetic acid, have been investigated in ethanol production by *S. stipitis*. It was demonstrated that 2.5 g/L acetic acid or 3 g/L furfural completely inhibited ethanol production.^14^ Under similar synthetic conditions, it was reported that 2.5 g/L acetic acid reduced ethanol production by 50% compared to the control, while up to 2 g/L furfural or 0.5 g/L HMF did not significantly affect fermentation.^25^ Another study found that 0.5 g/L furfural reduced *S. stipitis* proliferation by 79%, while 1.0 g/L acetic acid reduced it by 59% and 0.5 g/L HMF by 15%.^46^ These inhibitors can cause cellular metabolic damage, oxidative stress, and cytoplasmic acidification, which demand energy expenditure for proton extrusion.^47^

### 3.4 Fermentation of Hydrolysates Obtained from the EH of Untreated Corn Stover and Steam-Exploded Corn Stover

The combination of SE and EH is also considered environmentally friendly compared with other pretreatment and hydrolysis methods that use chemical agents such as acids. Therefore, to evaluate and compare different strategies for obtaining fermentable sugars and fermenting these hydrolysates from corn stover, EH assays were performed using both untreated and steam-exploded corn stover, followed by fermentation of the resulting sugars with *S. stipitis* (Table 6).

**Table 6:** Sugar Release and Ethanol Yields Obtained after EH of Untreated and Steam-Exploded Corn Stover Fermented by *Scheffersomyces stipitis*

pretreatment	FPU	glucose (g/L)	cellobiose (g/L)	xylose (g/L)	arabinose (g/L)	total sugars (g/L)	ethanol yield (g/L)	yield ethanol/sugar available (g/g)	yield ethanol/sugar consumed (g/g)^a^	theoretical yield based on sugar available (%)	theoretical yield based on sugar consumed (%)^a^
untreated	15	8.1 ± 0.5^c^	7.0 ± 0.4^a^	6.7 ± 2.4^a^	0.4 ± 0.0^b^	22.2 ± 2.6^b^	3.8 ± 0.2^c^	0.17 ± 0.03^b^	0.28 ± 0.07^b^	33.7 ± 5.7^b^	55.26 ± 13.96^b^
untreated	30	9.6 ± 0.1^c^	6.4 ± 0.2^a^	4.9 ± 0.1^a^	1.2 ± 0.0^a^	22.0 ± 0.3^b^	4.6 ± 0.2^c^	0.21 ± 0.00^b^	0.35 ± 0.00^a,b^	40.9 ± 0.8^b^	69.55 ± 0.59^a,b^
steam explosion	15	22.4 ± 0.6^b^	6.1 ± 0.2^a^	0.0 ± 0.0^b^	0.0 ± 0.0^c^	28.5 ± 1.0^a^	10.6 ± 1.0^b^	0.37 ± 0.05^a^	0.47 ± 0.06^a,b^	72.5 ± 9.5^a^	92.69 ± 12.21^a,b^
steam explosion	30	27.5 ± 1.2^a^	6.1 ± 0.1^a^	0.0 ± 0.0^b^	0.0 ± 0.0^c^	33.6 ± 1.1^a^	13.3 ± 0.1^a^	0.40 ± 0.01^a^	0.50 ± 0.04^a^	77.7 ± 1.8^a^	98.06 ± 6.92^a^

a Ethanol yield based on sugar consumed until the ethanol production peaks. Different letters in the same column indicate that the means differ significantly by the Tukey test (*p* ≤ 0.05).

When evaluating the sugar yield from the EH of untreated corn stover at 15 and 30 FPU, little variation was observed in the amount of total sugars released. The composition of the untreated corn stover showed 35.0 ± 0.0% cellulose, 26.3 ± 0.5% hemicellulose, and 18.1 ± 0.3% lignin. Thus, in 5 g of biomass, there were approximately 1.75 g of cellulose; using 30 FPU, it was possible to hydrolyze 85.7% (1.5 g) of the cellulose, with 58% of it in the form of glucose and 42% as cellobiose. The incomplete hydrolysis of cellulose, resulting in high cellobiose content, may be attributed to the recalcitrance of the untreated biomass. The release of xylose and arabinose can vary significantly depending on the genotype, maturity, and type of biomass, as these factors influence the hemicellulose composition,^48^ making it difficult to determine the precise amount of each monosaccharide that can be obtained. Thus, from 5 g of corn stover, approximately 1.3 g corresponded to hemicellulose, of which around 46.2% (0.6 g) was hydrolyzed into xylose and arabinose at both 15 and 30 FPU.

The corn stover treated by SE at 200 °C showed a composition of 74.1% cellulose, 2.6% hemicellulose, and 31.2% lignin. Thus, in 5 g of treated corn stover, approximately 3.7 g corresponds to cellulose. Using 30 FPU, it was possible to hydrolyze 83.8% of this fraction (3.1 g), with 80.6% released as glucose and 19.4% as cellobiose. Additionally, no xylose or arabinose was detected, as the SE pretreatment removed nearly all of the hemicellulose from the biomass. It is observed that the proportion of hydrolyzed cellulose was similar for both untreated and treated corn stover. However, in the treated biomass, most of the sugars were obtained in monomeric form (glucose). This is likely due to the SE depolymerizing lignin, removing hemicellulose, and exposing the cellulose fibers,^7^ thereby enhancing enzyme accessibility during hydrolysis.

The ethanol/sugar yield available (g/g) was significantly lower for the sugars obtained from the EH of untreated corn stover, as the resulting hydrolysate contained a lower proportion of glucose, which is the sugar that *S. stipitis* converts most efficiently into ethanol. As a result, ethanol yields compared to the theoretical maximum were 33.7 and 40.9% for 15 and 30 FPU, respectively. In contrast, ethanol production by *S. stipitis* from the sugars derived from the EH of pretreated corn stover showed much more favorable outcomes, reaching yields 130% to 250% higher than those from the untreated corn stover hydrolysate.

The ethanol/sugar availability of 0.37 g/g and 0.40 g/g and ethanol theoretical yields of 72.5 and 77.7%, observed in the fermentation of the enzymatic hydrolysates from pretreated corn stover, were slightly higher than those reported for the synthetic media fermentations in Table 2. These results indicate that the yeast has a strong ability to ferment in real complex media. Another factor that may help explain the better performance compared to synthetic media is the occurrence of simultaneous saccharification and fermentation since both the corn stover and cellulolytic enzymes remained present in the substrate during fermentation. This could have increased the availability of glucose during fermentation, which was immediately consumed by the yeast and, therefore, could not be quantified as part of the initial sugar concentration.

As shown in Table 6, ethanol yields calculated on the basis of sugar consumed provide important insights into the efficiency of fermentation following EH. For untreated corn stover, increasing enzyme loading from 15 to 30 FPU improved both sugar consumption and ethanol yield, resulting in ethanol yields based on sugar consumed of up to 0.35 g/g and theoretical efficiencies close to 70%. In steam-exploded substrates, the yield based on sugar available was substantially higher due to improved saccharification. Similarly, the yield based on sugar consumed also increased, reaching values close to 98%. This behavior is explained by the predominance of glucose among the sugars consumed, which exhibits a higher conversion efficiency to ethanol than pentoses, such as xylose and arabinose. This outcome is directly associated with the SE pretreatment, which effectively removes hemicellulose, thereby reducing the formation of C5 sugars and enhancing cellulose accessibility and digestibility, leading to a higher proportion of glucose released and subsequently converted into ethanol.

Figure 5 shows the sugar consumption and ethanol production profiles by *S. stipitis* using sugars derived from (a) untreated corn stover at 15 FPU, (b) untreated corn stover at 30 FPU, (c) steam-explosion pretreated corn stover at 15 FPU, and (d) pretreated corn stover at 30 FPU. In the assays using untreated corn stover, both glucose and xylose were completely consumed after 12 h of fermentation, coinciding with the peak of ethanol production. An increase in cellobiose concentration was observed at 12 h, suggesting that cellulolytic enzymes were still active during fermentation. Additionally, unlike the synthetic fermentations, cellobiose was not completely consumed.

**Figure 5:**
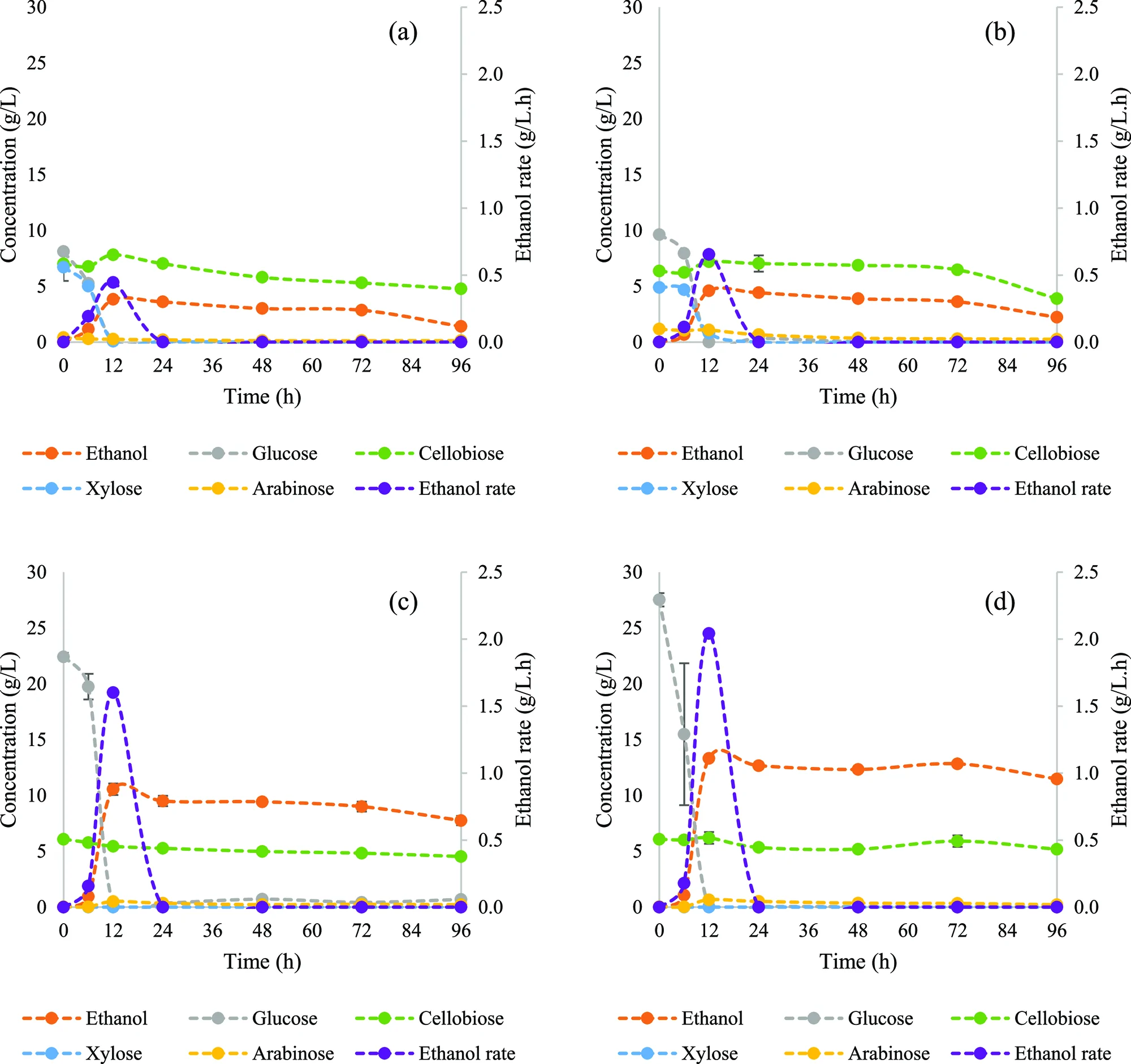
Fermentation profiles of hydrolysates obtained by EH of corn stover: (a) untreated material with 15 FPU; (b) untreated material with 30 FPU; (c) steam-exploded material with 15 FPU (SE + EH); and (d) steam-exploded material with 30 FPU (SE + EH).

The fermentation assays using hydrolysates obtained from pretreated corn stover also showed complete glucose consumption after 12 h of fermentation, coinciding with the peak ethanol yield at this time. Cellobiose was not fully consumed, decreasing from 6.1 g/L to 4.5 g/L with 15 FPU, increasing slightly from 6.1 g/L at 0 h to 6.2 g/L after 12 h, and then decreasing to 5.2 g/L after 96 h with 30 FPU. If the ethanol produced under the 15 FPU condition was derived solely from the initial glucose present at the start of fermentation, the ethanol/sugar yield (g/g) would be 0.47 g/g, and 0.48 g/g for the 30 FPU condition, values significantly higher than those reported in Table 2 for the synthetic substrate. This supports the idea that cellulolytic enzymes were still hydrolyzing cellulose simultaneously with fermentation, thereby increasing glucose availability and contributing to higher ethanol production.

Considering 12 h of fermentation, an ethanol production rate of 1.1 g/L.h is observed in Figure 5d, while between 6 and 12 h, a peak production rate of 2.04 g/L.h was recorded, this being the period of highest ethanol productivity in the fermentations of hydrolysates obtained via EH. From the EH of the sweet sorghum residue and co-fermentation with *S. cerevisiae* (MTCC 174) and *S. stipitis*, a yield of 16.8 g/L of ethanol and an ethanol/sugar yield of 0.47 g/g with a productivity of 0.26 g/L.h were obtained after 72 h of fermentation.^22^ Another study reported the fermentation of sugars derived from spent coffee grounds, where from 15.3 g/L glucose and 10.6 g/L mannose, an ethanol concentration of 11.6 g/L was achieved, resulting in an ethanol/sugar yield of 0.45 g/g and a productivity of 0.48 g/L.h after 24 h of fermentation.^49^

When comparing synthetic media experiments with those using hydrolysates obtained from biomass, it becomes evident that *S. stipitis* more efficiently assimilates glucose and converts it into ethanol. Xylose is the yeast’s second preferred sugar, leading to ethanol production at a lower proportion. Cellobiose appears to be the third preferred sugar, with some experiments showing that its presence reduced the ethanol yield that could otherwise be obtained from glucose (Figure 2 (A6)). In other cases, when all four sugars were present, there was evidence that cellobiose assimilation contributed to ethanol production (Figure 3 (A8)).

In fermentations with real hydrolysates obtained by SE and SWH of corn stover, cellobiose was almost completely consumed in most assays, likely due to the limited availability of alternative carbon sources (Figure 4). In contrast, when cellobiose was generated through EH, its assimilation was lower (Figure 5a,b), particularly when glucose was the only other carbon source available (Figure 5c,d). This may indicate that the presence of xylose favors the coassimilation of cellobiose by the yeast. Finally, arabinose was shown to be poorly assimilated in fermentations, and no ethanol production from this monosaccharide could be observed.

According to Table 7, ethanol yields obtained in this study are consistent with those reported for second-generation ethanol production using pentose-fermenting yeasts. While ethanol titers achieved here are generally lower than those reported for engineered *S. cerevisiae* strains and fed-batch processes, the ethanol yields based on consumed sugars (0.28–0.50 g/g) fall within the range commonly reported for *S. stipitis* fermentations of lignocellulosic hydrolysates. Similar yields have been observed for corn stover hemicellulosic hydrolysates (0.37–0.44 g/g) and sugarcane bagasse hydrolysates (0.41–0.43 g/g), indicating that the fermentative efficiency of *S. stipitis* in this study is comparable to that reported in the literature.

**Table 7:** Comparison between Different Studies for Second-Generation Ethanol Production^a^

study	substrate and process	yeast	ethanol (g/L)	yield ethanol (g/g sugar)	productivity (g/L·h)
^50^	corn stover hemicellulose (acid hydrolysis)	*S. stipitis* CBS 6054	10–15	0.37–0.44	0.24–0.48
 ^51^	corn stover hydrolysate (alkaline pretreatment and EH)	*S. stipitis* + *Z. mobilis*	13–19	0.28–0.38*	0.19–2.18
 ^10^	corn stover (SE e EH)	S. cerevisiae (engineered strain)	22.96 (fed-batch 51.12)	0.45 (0.44 fed-batch)	0.71
 ^52^	vineplant waste hydrolysate (acid hydrolysis)	yeast strains	2–5	0.2–0.43*	—
^18^	sugarcane bagasse hydrolysate (sulfite + SE and EH)	*S. stipitis* CBS 5773	9–10	0.41–0.43	1.2–1.3
 ^41^	sugarcane bagasse hydrolysate (SWH and EH)	*S. cerevisiae* SA-1 + *S. stipitis* NRRLY-7124	2.2	0.32	0.17
 ^9^	soybean straw hydrolysate (diluted and supplemented with sugar, SWH)	*Wickerhamomyces* sp. UFFS-CE-3.1.2	0.69–6.11	0.19–0.50	0.06
this study	corn stover (SE and SWH)	*S. stipitis* ATCC 58376	0.26–0.50	0.03–0.100.12–0.28*	0.01–0.05
this study	corn stover (SE and EH)	*S. stipitis* ATCC 58376	3.8–13.3	0.17–0.400.28–0.50*	0.32–1.11

a Yields marked with an asterisk correspond to ethanol yield calculated based on sugar consumed.

The lower ethanol concentrations observed are primarily associated with limited sugar availability and incomplete sugar consumption, particularly in hydrothermal hydrolysates rather than with metabolic constraints of ethanol formation. Possibly because SE and SWH are not selective processes (unlike enzymes), parallel reactions occur during hydrolysis, leading to the formation of inhibitory compounds derived from the degradation of cellulose, hemicellulose, and lignin. These compounds can hinder sugar assimilation by the yeast even when fermentable sugars are present in the hydrolysate. This behavior highlights the need for further process optimization aimed at increasing fermentable sugar concentrations while minimizing inhibitor formation as well as the implementation of detoxification strategies to improve overall fermentation performance.

In contrast, studies reporting higher ethanol titers typically rely on genetically engineered strains, fed-batch strategies, or sugar supplementation, which are not directly comparable to the batch fermentations of nondetoxified hydrolysates evaluated here. Therefore, the results of this work provide a realistic assessment of fermentation performance under inhibitory conditions and highlight the importance of distinguishing between the ethanol yield and ethanol titer when evaluating second-generation ethanol processes.

## 4 Conclusion

This study demonstrated that ethanol production from lignocellulosic hydrolysates is strongly dependent on both the processing route and the resulting substrate composition. Hydrothermal processes, such as SE and SWH, resulted in low ethanol yields because of limited sugar concentrations and the presence of inhibitory compounds. In contrast, EH, particularly when combined with SE pretreatment, produced substrates with higher sugar availability and minimal inhibitor formation, leading to a significantly improved ethanol production.

More importantly, the combined analysis of ethanol yields based on total sugar available and sugar consumed revealed that low overall yields are primarily associated with limitations in sugar uptake rather than the intrinsic metabolic inefficiency of *S. stipitis*. Even under inhibitory conditions, the yeast maintained a relatively high conversion efficiency for the fraction of sugars assimilated.

These findings highlight that improving ethanol production from lignocellulosic biomass should focus not only on increasing sugar release but also on enhancing sugar accessibility and mitigating inhibition effects that restrict the microbial assimilation. The dual-yield analytical approach proposed in this study provides a robust framework for identifying process bottlenecks and guiding the development of more-efficient second-generation ethanol production strategies.
